# Toxic myocarditis presenting as an acute coronary syndrome

**DOI:** 10.1186/s43055-022-00923-9

**Published:** 2022-11-17

**Authors:** Tushar Kalekar, Reetika Kapoor, Nikhith Soman, Tejvir Singh, Karthik Mohanan

**Affiliations:** Radiodiagnosis department, Dr D.Y. Patil Medical College and Research Centre, Sant Tukaram Nagar, Pimpri Chinchwad, Pune, Maharashtra India 411018

**Keywords:** Myocarditis, ST elevation, Marijuana, Cannabis, Cardiac MRI

## Abstract

**Background:**

Acute myocarditis is one of the causes of acute non-ischemic myocardial injury mimicking acute coronary syndrome (ACS) on presentation. It is usually underdiagnosed due to the non-specificity of presenting symptoms, elevated troponin levels, and abnormal ECG (electrocardiogram) findings. Delayed contrast-enhanced cardiac MRI (CMR) is known as the gold standard imaging modality for differentiating acute non-ischemic myocardial injury from infarcted myocardium. The most frequent cause of myocarditis is viral infections, but further infrequent causes include other infectious pathogens, toxins, hypersensitivity drug reactions, and autoimmune diseases. Cannabis is one of the most abused illicit and recreational drugs in the world among adolescents and adults. Also, many reports of marijuana-associated cardiovascular risks have been established previously, mainly presenting as arrhythmias, myocardial infarction, and myocarditis.

**Case presentation:**

A 19-year-old female, complaining of radiating chest pain to the left arm and jaw, along with associated sweating for 1 day. Initial work-up showed elevated troponin levels with ST segment elevation on ECG. On further investigation, CMR showed findings of myocarditis and negative virology work-up led to a work-up of proper history, which revealed marijuana abuse, with a history of consumption 3 days before presentation. Hence, it was diagnosed as toxic myocarditis secondary to presumed cannabis abuse and treated for same.

**Conclusions:**

Non-ischemic myocardial injury causes like myocarditis should be considered in young patients especially, who are presenting to emergency with ACS. CMR should be used as a first line diagnostic imaging modality (based on its availability) in cases mimicking an ACS or suspected myocarditis, especially in young patients. Though viral etiology is considered the most common cause of myocarditis, less common causes such as toxic myocarditis must be considered in cases of young individuals presenting with ACS in an emergency.

## Background

Acute myocarditis presents with varied symptoms extending from mild dyspnea or chest pain to cardiogenic shock and finally death. Most common causes are viral infections, whereas some of the less common being other pathogens, giant-cell myocarditis, hypersensitivity drug reactions or toxic, and sarcoidosis [1]. The vast majority of patients show non-specificity in presentation showcasing acute coronary syndrome (ACS) like symptoms, elevated troponin, and abnormal ECGs; thus, making a diagnosis of myocarditis challenging and is often a disease of exclusion [2]. Contrast-enhanced cardiac MRI(CMR) and echocardiography; mainly transthoracic echocardiography (TTE) are used as a standard noninvasive imaging tool in patients with suspected myocarditis [3, 4]. Toxic myocarditis is seen in abuse of drugs like cannabis, which is the most abused illicit substance in India and the world [5]. Cardiovascular adverse effects of cannabis broadly range from tachycardia, arrhythmias, hypotension, hypertension, ACS, myocardial infarction, myopericarditis to cardiomyopathy leading to cardiac death [6, 7]. Few proven cases of myocarditis after marijuana abuse have been reported till date [8–13]; however, many of these cases lack definitive toxicologic and pathologic data. We hereby describe an unusual case of myocarditis, mimicking an acute coronary syndrome in a young female patient, presumed secondary to drug abuse.

## Case presentation

**Fig. 1:**
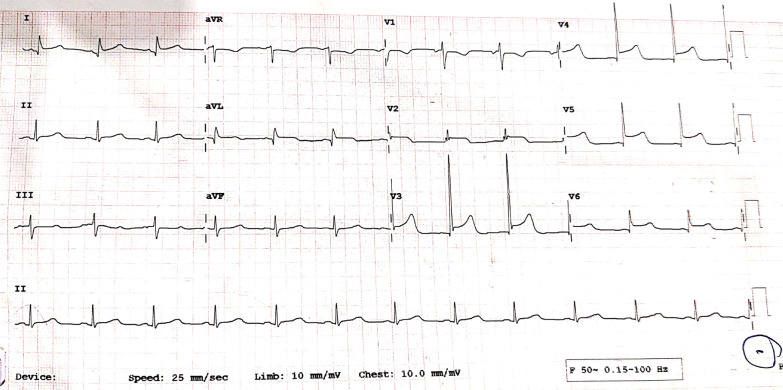
ECG at initial presentation—showing ST segment elevation in the leads I, aVL, V4, V5, and V6 leads

**Fig. 2:**
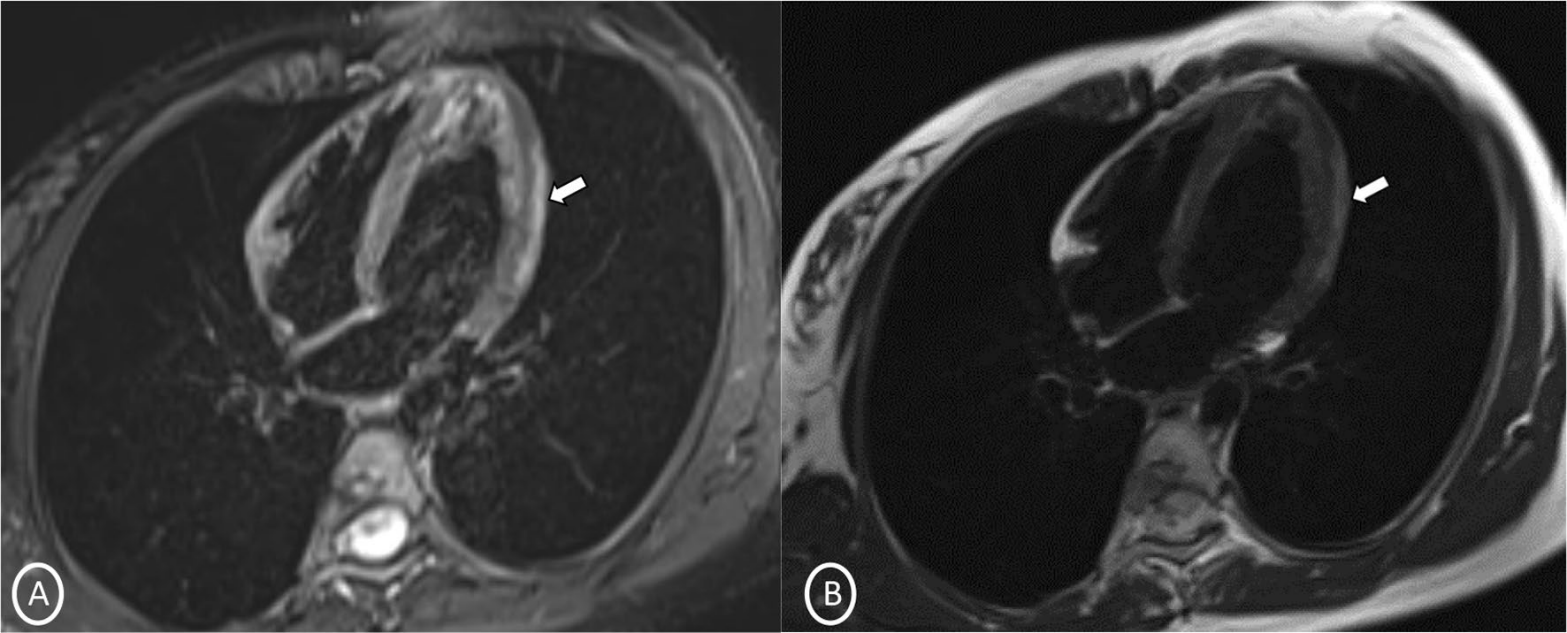
Long-axis 4 chambers **A** T2 STIR Dark blood; **B** T2 Dark blood—showing patchy T2 hyperintensity in subepicardial region along left lateral wall

**Fig. 3:**
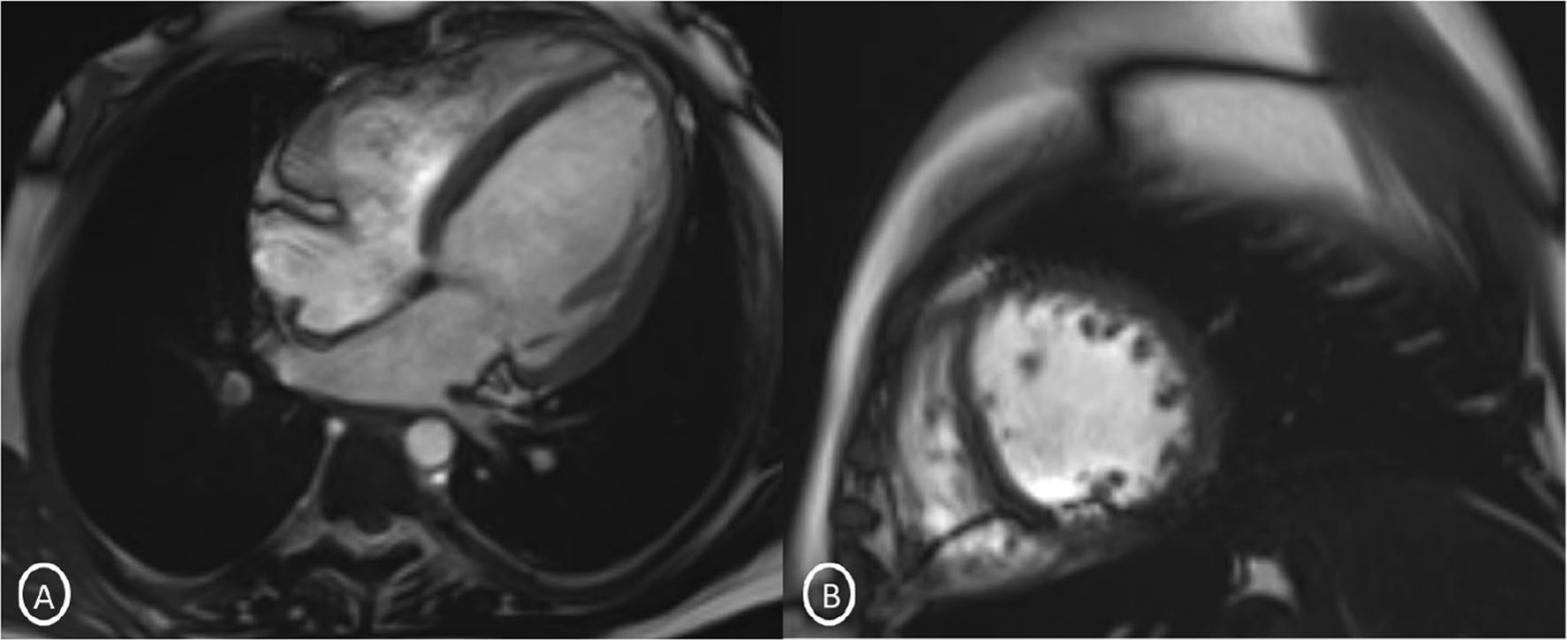
Snapshot from CINE Image T2 **A** Long-axis 4 chambers; **B** short-axis 2 chambers—showing mild global hypokinesia and left ventricle dilatation

**Fig. 4:**
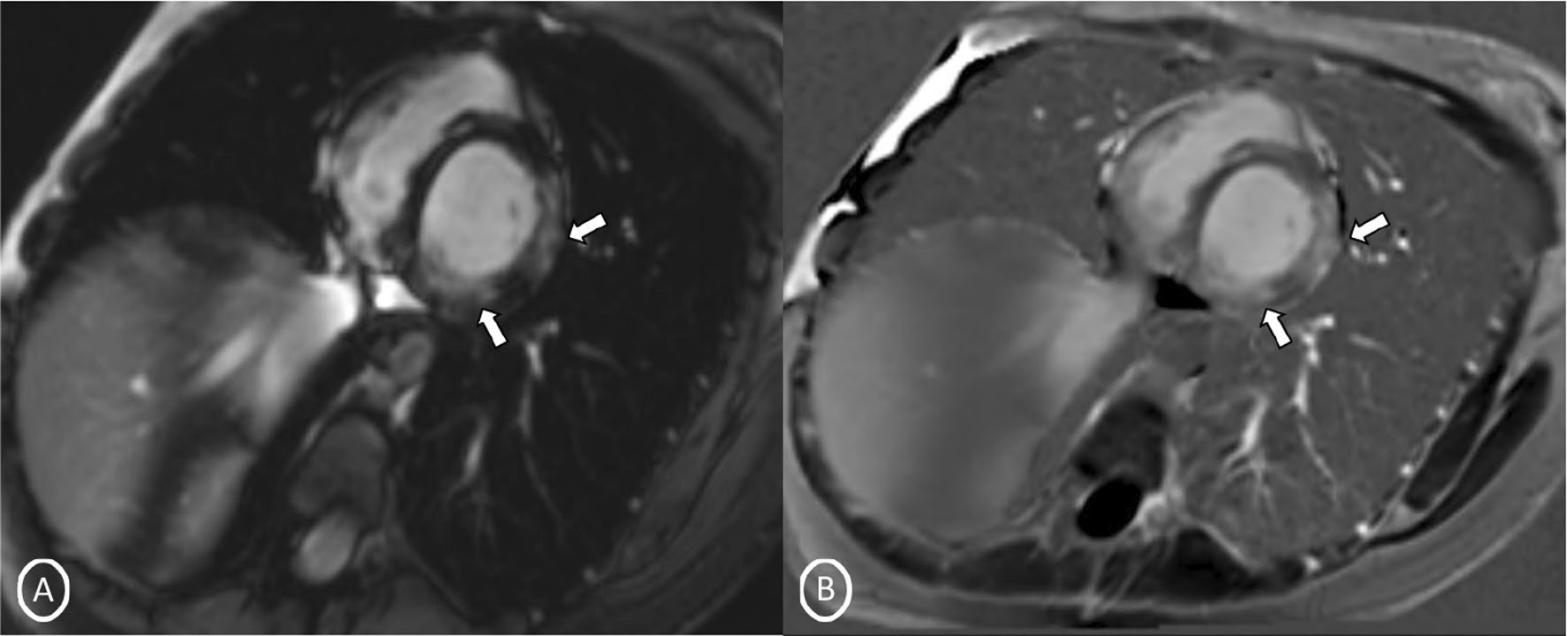
Short-axis 2-chamber PSIR sequence (basal cavity) **A** MAG; B PHASE images—showing patchy delayed gadolinium enhancement of intramural, subepicardial, tans-mural enhancement in inferior and lateral wall segments of cardiac basal cavity

**Fig. 5:**
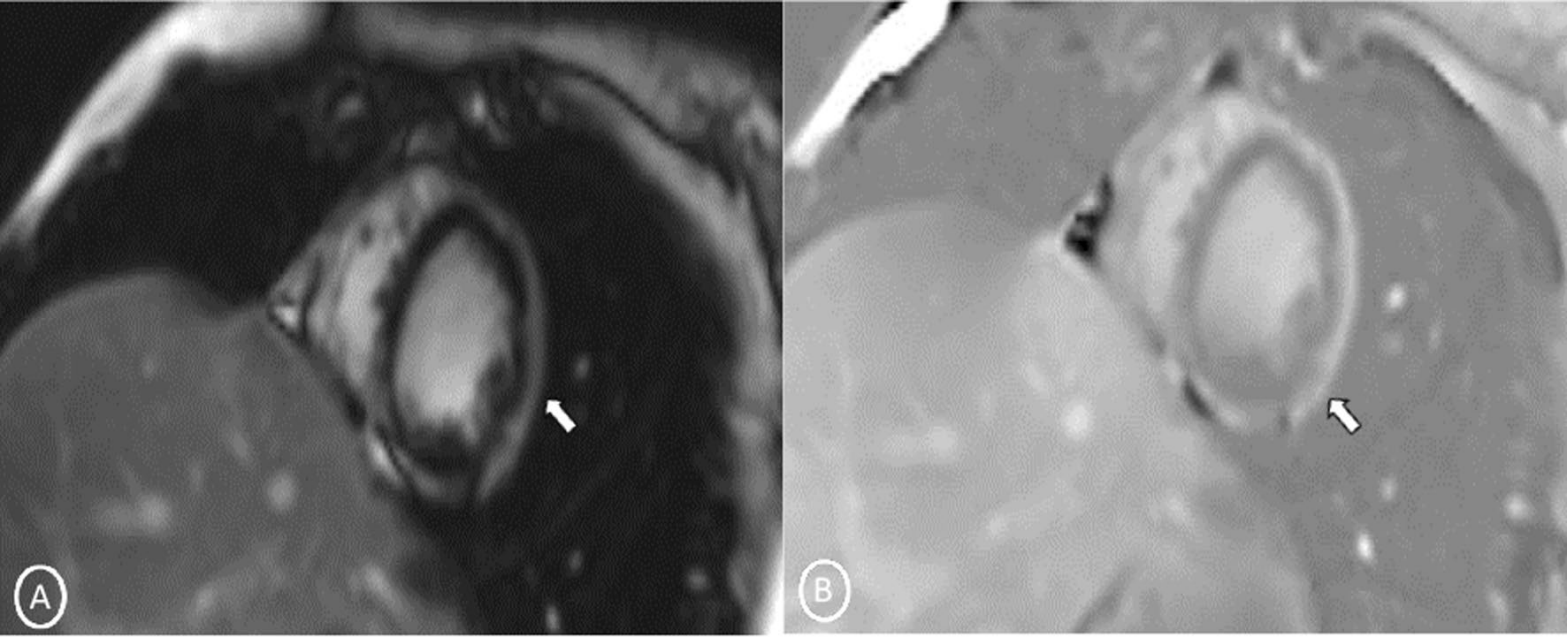
Short-axis 2-chamber PSIR sequence (Mid Cavity)- **A** MAG; **B** PHASE images—showing patchy delayed gadolinium enhancement of subepicardial in multiple segments of cardiac mid cavity

**Fig. 6:**
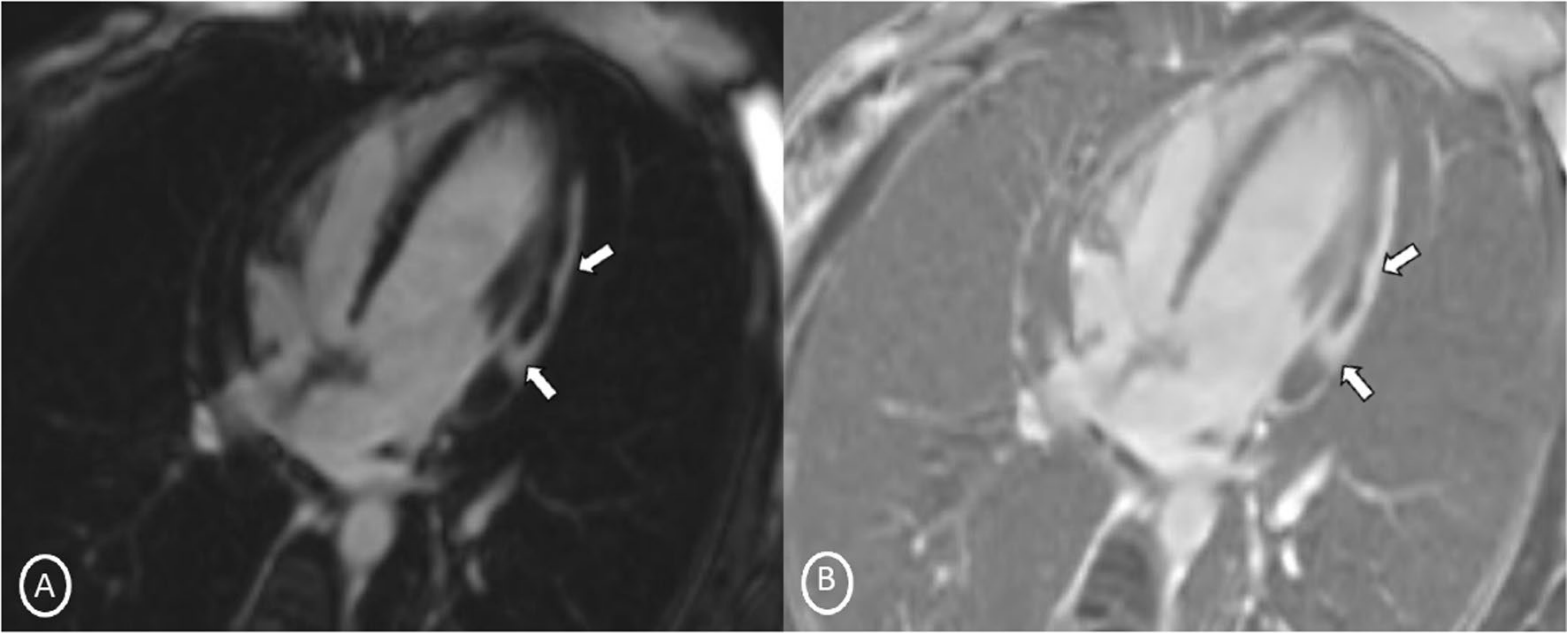
Long-axis 4-chamber PSIR sequence—**A** MAG; **B** PHASE images—showing patchy delayed gadolinium enhancement of intramural, subepicardial as all tans-mural enhancement along the left lateral wall

A 19-year-old female had presented to our Emergency Department, complaining of sudden severe chest and epigastric pain since 1 day, which was associated with sweating. The pain was radiating to neck and left arm. The pain did not relieve on rest but subsided on medications. Similar episodes were also reported in the past few days, however, with less severity. The patient confirmed to not having shortness of breath, palpitation, syncope, dizziness, nausea, emesis, fever, or cough. There was no history of any previous cardiac ailment/surgery/other identifiable cardiovascular risk factors or any other history of infection. There is no evidence to suggest chest wall trauma. There is no history of comorbidities or any history of smoking. There is no significant family history or any similar past history. Upon admission, patient was conscious, alert with heart rate of 86 bpm, blood pressure measuring 110/60 mm Hg, 97% oxygen saturation at room air, temperature of 36.4 °C, and respiration rate of 15 breaths/min. The cardiac biomarker troponin T levels were extremely raised, measuring 50,000 pg/ml, and CK-MB was raised, measuring 111U/Lt. Laboratory findings showed that inflammation markers were normal, C-reactive protein (5.8.0 mg/L) and D-dimer (94 ng/mL). Other laboratory findings such as lipid profile, complete blood count, electrolytes, fasting blood glucose, HBA1c, and renal and liver function tests were normal. Cardiac auscultation showed normal S1, S2 without murmurs or pericardial rub and any sign of heart failure. Initial ECG revealed ST segment elevation in the leads I, aVL, V4, V5, and V6 leads, normal sinus rhythm (60 beats per min) without any other abnormalities (Fig. 1), which was resolved by day 3. Arrhythmias were not found during continuous cardiac monitoring. Echocardiogram showcased an ejection fraction of the left ventricle to be 60%, whereas the echocardiographic metrics were not very significant. Patient was then treated as ACS with a salicylate-based nonsteroidal anti-inflammatory drug (NSAID), anticoagulants, and anti-thrombolytics. CMR was done on 3 T MRI machine with acquisition including T2/STIR Dark blood four-chamber long axis; CINE images in short axis, long axis, RVOT, and LVOT; and early dynamic gadolinium enhancement images and delayed gadolinium enhancement in PSIR images. T2/STIR Dark blood sequences showed T2 hyperintensity in the subepicardial region along left lateral wall (Fig. 2A, B). There was associated mild global hypokinesia with mildly reduced ejection fraction/systolic function (Fig. 3A, B). Patchy delayed gadolinium enhancement of intramural, subepicardial as all tans-mural enhancement with non-involvement of the subendocardium (suggesting a non-ischemic pattern) in multiple segments of cardiac basal (Fig. 4A, B), mid cavity (Fig. 5A, B), and apical cavity—with predominant involvement of lateral (Fig. 6A, B) and inferior wall segments in the basal cavity. There were no signs of infarction of myocardium; therefore, the patient was diagnosed with acute myocarditis (based on Lake Louise Criteria for Acute Myocarditis). Further, viral and autoimmune workups results were found to be negative. It included hepatitis (B, C), human immunodeficiency virus, Coxsackie virus, COVID-19, cytomegalovirus, Epstein–Barr virus (EBV), and Parvovirus B-19. On further analysis and detailed relevant history workup, the patient admitted to using cannabis for the past 1 year with last exposure being 3 days before admission. On the basis of detailed clinical history, findings on CMR with other negative viral markers, and possible etiological factors, a final diagnosis of myocarditis presumed secondary to marijuana/cannabis toxin abuse was made. Patient was released from the hospital on the seventh day with advised conservative treatment. The patient was counseled to abstain completely from cannabis or related drugs. Patient was advised to repeat a CMR control, three months later. A 3–4-month restriction on high-intensity activity was also given to the patient following discharge.

## Discussion

Myocarditis refers to inflammation of the myocardium with incidence ranging from 1 to 9% in routine autopsies and about 5–12% in autopsy cases of sudden cardiac death. Based on the clinical and pathologic manifestations, myocarditis is divided into four groups: fulminant, acute, chronic active, and chronic [4, 14]. Myocarditis presents with an extended range of symptoms and is often non-diagnosed underlying etiology of chronic dilated cardiomyopathy and acute heart failure, leading to sudden death [15]. Definitive diagnosis of myocarditis is by histology. Myocarditis presents with wide range of symptoms ranging from mild dyspnea or chest pain subsiding without specific treatment, dyspnea, palpitations, fatigue, dizziness, or syncope to even cardiogenic shock leading to death. Dilated cardiomyopathy with chronic heart failure is a long-term sequela of myocarditis [15]. Acute myocarditis mimics an ACS on initial presentation with elevated troponin, abnormal ECGs including ST segment elevation, AV block, supraventricular or ventricular arrhythmia, and echocardiography often showing preserved left ventricular function; except in severe cases, mild regional dysfunction/localized wall motion abnormalities (WMAs) can be seen; thus, diagnosis of myocarditis becomes challenging and is often a disease of exclusion from other cardiac pathologies, because of non-specificity in presentation [2, 4]. The most frequent cause of myocarditis is viral infections, but other infectious pathogens such as fungi, bacteria, protozoans, as well as toxins hypersensitivity drug reactions, giant-cell myocarditis, autoimmune diseases, and sarcoidosis can also cause myocarditis [1, 15]. In patients with suspected myocarditis, echocardiography, mainly transthoracic echocardiography (TTE) and CMR, serves as standard noninvasive imaging modality which preclude invasive procedures such as endomyocardial biopsy and coronary angiography. Nuclear imaging has been less sensitive to diagnose myocarditis [3, 4].

Due to its high spatial resolution and with just different protocol components—steady-state free precession; T2-weighted triple inversion recovery; and phase-sensitive inversion-recovery gradient echo (early and delayed Gd enhancement), cardiovascular MRI gives detailed information on myocardial tissue characteristics, even subtle, patchy areas of in vivo inflammatory hyperemia, edema, necrosis/fibrosis (Lake Louise Criteria for Acute Myocarditis); contractile dysfunction, and accompanying pericardial effusion [3, 16]. CMR can distinguish between variable causative etiologies like myocarditis, acute infarction, and other cardiomyopathies. Thus, delayed CMR is known as the gold standard for differentiating acute non-ischemic myocardial injury and in vivo detection of scarring associated with myocardial infarction [17] and also helps in evaluating the myocarditis’s activity and severity. The conventional findings of myocarditis are delayed epicardial enhancement and sparing the subendocardium, with non-segmental distribution. Therefore, delayed CMR provides comprehensive and early diagnosis in patients with suspected myocarditis, thus aiding in appropriate treatment and also in cases of follow-up [3, 16]. Thus, CMR has better accuracy in detecting subtle changes compared to TTE which helps in assessing the functional injury [4]. Varied illicit and licit drugs like anticonvulsants, antipsychotics, antibiotics, alcohol, amphetamines, and cannabis have been implicated as causes of myocarditis [18]. Cannabis sativa commonly known as Marijuana is one of the most used recreational drugs and the most abused illicit substance in India and the world [5]. As per Ministry of Social Justice and Empowerment’s survey conducted to understand the magnitude of substance use in India, 2.83% or 31 million people aged 10–70 years were found to be actively consuming cannabis products in 2019 [19]. Despite few studies stating the medical use of marijuana in treatment of neuropathic pain and certain cannabinoids in treating nausea and vomiting from cancer chemotherapy, the abuse of marijuana has been associated with adverse psychiatric, neurological, respiratory, gastrointestinal and cardiovascular effects [20]. Two of the major active cannabinoids in marijuana are tetrahydrocannabinol (THC) and cannabidiol (CBD), which act through various widely spread CB receptors in the body [6]. Marijuana shows cardiovascular adverse effects by marijuana, which are mainly caused via action of THC on CB1 receptors present in myocytes and range from rhythm and rate irregularities (like tachycardia and arrhythmias), blood pressure changes (like hypotension or hypertension), atherosclerosis, vasospasm (leading to acute coronary syndrome), vasodilation, myocardial infarction, coronary dissection, myopericarditis to cardiomyopathy leading to cardiac death [6, 7, 21]. The exact mechanism responsible for the myocarditis is unclear; however, presence of adulterants or contaminants like heavy metals and pesticides is considered a possible hypothesis [12]. Myocarditis secondary to cannabis is seen in few proven cases reported till date [8–13]. In 2008, the first case of a 29-year-old man who developed toxic myocarditis from cannabis addiction and was maintained for 96 days by a left ventricular assist device was published by Leontiadis et al. [22]. In our case, as patient presented with sudden onset of severe chest and epigastric pain, radiating to neck and left arm and associated sweating with raised troponin levels and abnormal ECG showing ST elevation, the diagnostic picture appeared to be more like ACS/ myocardial infarction. However, on further imaging findings on CMR, the diagnosis of myocarditis was made, which after detailed revaluation of clinical history was presumed to be secondary to toxin (cannabis) abuse. Thus, non-ischemic myocardial injury causes like myocarditis should be considered in young patients especially, who are presenting to emergency with symptoms mimicking ACS, and thus, CMR should be advised as the first line of diagnostic imaging tool (based on its availability) in all such cases. Although viral etiology is the most frequent cause of myocarditis, however, in a patient with negative virological markers, a detailed history pertaining to the drug use/abuse should be done and toxin-related myocarditis should be considered as a possible etiology. Also, many studies have shown the association between marijuana and myocarditis, so in our case with the history of marijuana abuse which was aided by diagnosis of myocarditis on CMR, a diagnosis of toxic myocarditis presumed secondary to marijuana/cannabis toxin abuse was considered.

## Conclusions

We report here an unusual case of myocarditis with symptoms of an acute coronary syndrome, presumed secondary to toxin (cannabis). The fact that the patient was diagnosed only after CMR imaging findings and detailed proper drug history highlights the importance of adding CMR as a first-line diagnostic imaging tool (based on its availability) by physicians and cardiologists in cases mimicking an ACS or suspected myocarditis, which might get undiagnosed in routine investigations, more so in the young population. Therefore, non-ischemic myocardial injury causes like myocarditis should be considered in young patients especially, who are presenting to emergency with ACS. Also, drugs/toxins should be considered as an important etiological cause of myocarditis in young individuals. Cannabis has been linked to myocarditis, cardiovascular problems such arrhythmia, sudden cardiac death, coronary artery vasospasm, and myocardial infarction, according to prior literature. Hence, cannabis-induced myocarditis is a rare, underdiagnosed cause of myocarditis as it has a low level of suspicion and non-specific symptomatic presentation. Based on this case, we think it is important to look at any toxins or cannabis usage in young patients who have been diagnosed with myocarditis syndrome.

## Data Availability

All data generated or analyzed during this study are included in this published article.

## Abbreviations

ACS: Acute coronary syndrome
ECG: Electrocardiogram
MRI: Magnetic resonance imaging
CMR: Contrast-enhanced cardiac MRI
TTE: Transthoracic echocardiography
HBA1c: Hemoglobin A1c
CK-MB: Creatine kinase-MB
CBD: Cannabidiol
THC: Tetrahydrocannabinol
PSIR: Phase-sensitive inversion-recovery gradient echo
MAG: Magnitude
